# Carmustine-Induced Phosphatidylserine Translocation in the Erythrocyte Membrane

**DOI:** 10.3390/toxins5040703

**Published:** 2013-04-19

**Authors:** Kashif Jilani, Florian Lang

**Affiliations:** Department of Physiology, University of Tuebingen, Gmelinstr. 5, Tuebingen D-72076, Germany; E-Mail: kashif_cbc@yahoo.com

**Keywords:** phosphatidylserine, carmustine, calcium, cell volume, eryptosis

## Abstract

The nitrosourea alkylating agent, carmustine, is used as chemotherapeutic drug in several malignancies**.** The substance triggers tumor cell apoptosis. Side effects of carmustine include myelotoxicity with anemia. At least in theory, anemia could partly be due to stimulation of eryptosis, the suicidal death of erythrocytes, characterized by cell shrinkage and breakdown of phosphatidylserine asymmetry of the cell membrane with phosphatidylserine exposure at the erythrocyte surface. Stimulators of eryptosis include increase of cytosolic Ca^2+^ activity ([Ca^2+^]_i_). The present study tested whether carmustine triggers eryptosis. To this end [Ca^2+^]_i_ was estimated from Fluo3 fluorescence, cell volume from forward scatter, phosphatidylserine exposure from annexin V binding, and hemolysis from hemoglobin release. As a result a 48 h exposure to carmustine (≥25 µM) significantly increased [Ca^2+^]_i_, decreased forward scatter and increased annexin V binding. The effect on annexin V binding was significantly blunted in the absence of extracellular Ca^2+^. In conclusion, carmustine stimulates eryptosis at least partially by increasing cytosolic Ca^2+^ activity.

## 1. Introduction

Carmustine (1,3-bis-(2-chloroethyl)-1-nitrosourea), a nitrosourea alkylating agent is widely used for the treatment of malignancies [1,2,3,4,5,6,7]. As systemic administration of nitrosoureas was poorly effective in the treatment of high grade glioma, carmustine wafers have been developed which deliver high local concentrations of the drug [1]. Carmustine is mainly effective by alkylating DNA and RNA [1] and inducing apoptosis [2,8,9,10,11,12]. Mechanisms involved in the triggering of apoptosis by carmustine include oxidative stress [2,8] at least in part by inhibition of glutathion reductase [13,14]. Carmustine induced oxidative stress is at least partially effective by increasing Ca^2+^ entry from extracellular space [15]. Side effects of systemic carmustine administration include anemia [2,8], which may at least partially result from erythrocyte death.

Suicidal erythrocyte death or eryptosis is characterized by erythrocyte shrinkage and breakdown of phosphatidylserine (PS) asymmetry of the erythrocyte cell membrane [16,17]. Stimulators of eryptosis include increase of cytosolic Ca^2+^ activity ([Ca^2+^]_i_), which may result from Ca^2+^ entry through Ca^2+^ permeable cation channels [18,19]. The increase of [Ca^2+^]_i _results in cell shrinkage due to activation of Ca^2+^ sensitive K^+^ channels [20], K^+^ exit, hyperpolarization, Cl^- ^exit and thus cellular loss of KCl with osmotically obliged water [21]. The increase of [Ca^2+^]_i _further leads to breakdown of PS asymmetry of the erythrocyte cell membrane with translocation of PS to the erythrocyte surface [22]. The Ca^2+^ sensitivity of eryptosis is enhanced by ceramide [23]. Additional stimulators of eryptosis include energy depletion [24], caspase activation [25,26,27,28,29] and dysregulation of AMP activated kinase AMPK [19], cGMP dependent protein kinase [30], Janus activated kinase JAK3 [31], casein kinase [32,33], p38 kinase [34], PAK2 kinase [35] as well as sorafenib [36] and sunitinib [37] sensitive kinases.

Eryptosis is stimulated by a myriad of xenobiotics [37,38,39,40,41,42,43,44,45,46,47,48,49,50,51,52,53,54,55,56,57,58,59,60,61,62,63,64,65,66,67,68] and excessive eryptosis participates in the pathophysiology of several clinical disorders [16], such as diabetes [29,69,70], renal insufficiency [71], hemolytic uremic syndrome [72], sepsis [73], malaria [74,75,76,77,78], sickle cell disease [79], Wilson’s disease [77], iron deficiency [80], malignancy [81], phosphate depletion [82], and metabolic syndrome [64].

The present study explored the effect of carmustine on erythrocyte [Ca^2+^]_i_, cell volume and PS exposure at the cell surface. As a result, carmustine increases [Ca^2+^]_i_, decreases erythrocyte volume and enhances the PS abundance at the erythrocyte surface. 

## 2. Results and Discussion

**Figure 1:**
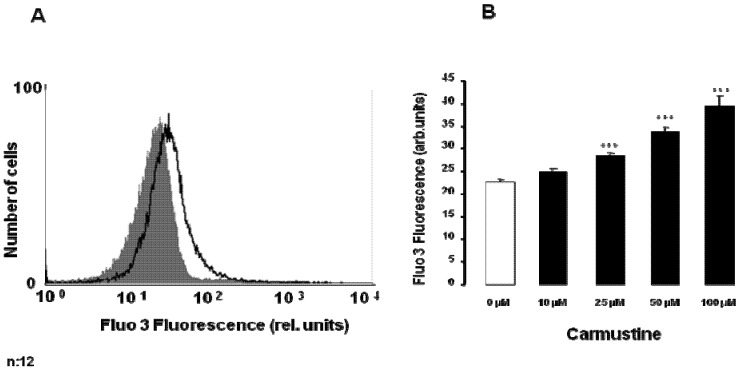
Effect of carmustine on erythrocyte cytosolic Ca^2+^ concentration. (**A**) Original histogram of Fluo3 fluorescence in erythrocytes following exposure for 48 h to Ringer solution without (grey shadow) and with (black line) presence of 100 µM carmustine; (**B**) Arithmetic means ± SEM (*n* = 12) of the Fluo3 fluorescence (arbitrary units) in erythrocytes exposed for 48 h to Ringer solution without (white bar) or with (black bars) carmustine (10–100 µM). *** (*p* < 0.001) indicates significant difference from the absence of carmustine (ANOVA).

The present study was designed to explore whether carmustine stimulates eryptosis, the suicidal death of erythrocytes. As eryptosis is triggered by increase of cytosolic Ca^2+^ activity ([Ca^2+^]_i_), Fluo3 fluorescence was employed to estimate [Ca^2+^]_i_. To this end, the erythrocytes were incubated in Ringer solution without or with carmustine (10–100 µM), loaded with Fluo3 AM and Fluo3 fluorescence quantified by FACS analysis. As illustrated in Figure 1, a 48 hours exposure of human erythrocytes to carmustine was followed by an increase of Fluo3 fluorescence, an effect reaching statistical significance at 25 µM carmustine concentration. Thus, carmustine treatment was followed by increase of [Ca^2+^]_i_ in human erythrocytes.

**Figure 2:**
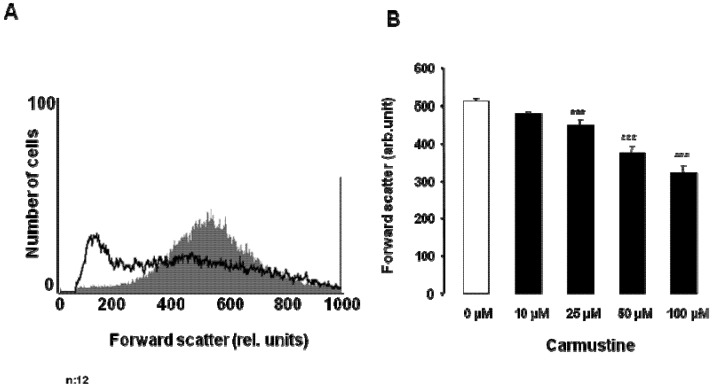
Effect of carmustine on erythrocyte forward scatter. (**A**) Original histogram of forward scatter of erythrocytes following exposure for 48 h to Ringer solution without (grey shadow) and with (black line) presence of 100 µM carmustine; (**B**) Arithmetic means ± SEM (*n* = 12) of the normalized erythrocyte forward scatter (FSC) following incubation for 48 h to Ringer solution without (white bar) or with (black bars) carmustine (10–100 µM). *** (*p* < 0.001) indicates significant difference from the absence of carmustine (ANOVA).

An increase of [Ca^2+^]_i_ is expected to activate Ca^2+^ sensitive K^+^ channels leading to cellular loss of KCl together with osmotically obliged water and thus to cell shrinkage. Accordingly, cell volume was estimated from forward scatter in FACS analysis. As shown in Figure 2, a 48 h treatment with carmustine resulted in a decrease of forward scatter, an effect reaching statistical significance at 25 µM carmustine concentration. Accordingly, carmustine treatment was followed by erythrocyte shrinkage.

**Figure 3:**
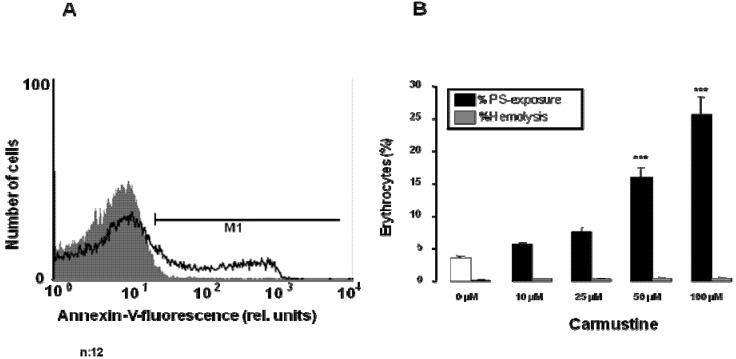
Effect of carmustine on PS exposure and hemolysis. (**A**) Original histogram of annexin V binding of erythrocytes following exposure for 48 h to Ringer solution without (grey shadow) and with (black line) presence of 100 µM carmustine; (**B**) Arithmetic means ± SEM (*n* = 12) of erythrocyte annexin V binding following incubation for 48 h to Ringer solution without (white bar) or with (black bars) presence of carmustine (10–100 µM). For comparison, arithmetic means ± SEM (*n* = 4) of the percentage of hemolysis is shown as grey bars. *** (*p* < 0.001) indicates significant differences from the absence of carmustine (ANOVA).

An increase of [Ca^2+^]_i_ is further expected to trigger cell membrane scrambling with breakdown of PS asymmetry of the cell membrane and appearance of phosphatidsylserine at the cell surface. Accordingly, PS abundance at the cell surface was estimated utilizing annexin V binding in FACS analysis. As shown in Figure 3, a 48 h carmustine treatment increased the percentage of annexin V binding erythrocytes, an effect reaching statistical significance at 50 µM carmustine concentration. Accordingly, carmustine triggered cell membrane scrambling.

In a separate series of experiments, hemolysis was estimated by determination of hemoglobin in the supernatant. As illustrated in Figure 3, the percentage of hemolyzed erythrocytes tended to increase slightly following exposure of erythrocytes for 48 h to carmustine, an effect, however, not reaching statistical significance up to 100 µM carmustine concentration (Figure 3). In any case, the percentage of hemolyzed erythrocytes remained one magnitude lower than the percentage of erythrocytes exposing PS.

**Figure 4:**
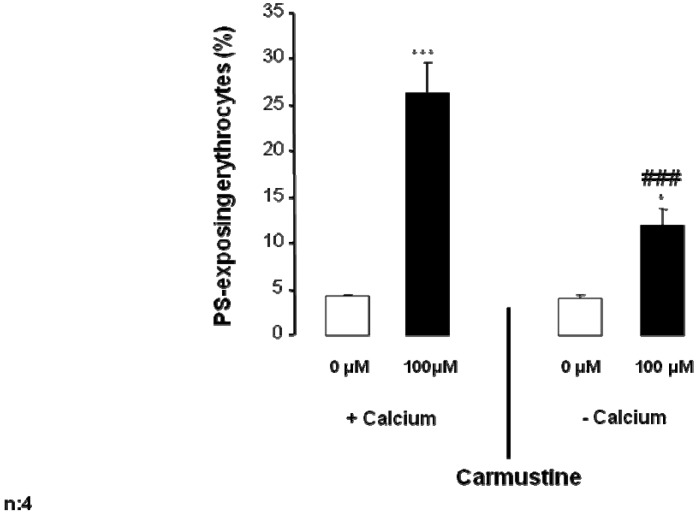
Effect of Ca^2+^ withdrawal on carmustine induced annexin V binding. Arithmetic means ± SEM (*n* = 4) of the percentage of annexin V binding erythrocytes after a 48 h treatment with Ringer solution without (white bar) or with (black bars) 100 µM carmustine in the presence (left bars, +Calcium) and absence (right bars, −Calcium) of calcium. * (*p* < 0.05), *** (*p* < 0.001) indicate significant difference from the absence of carmustine (ANOVA), ### (*p* < 0.001) indicates significant difference from the respective values in the presence of Ca^2+^.

Further experiments were performed to test whether the stimulation of cell membrane scrambling following carmustine treatment was partially or even fully explained by Ca^2+ ^entry from the extracellular space. To this end, erythrocytes were exposed to 100 µM carmustine for 48 h in either the presence of 1 mM extracellular Ca^2+^ or in the absence of extracellular Ca^2+^ and presence of the Ca^2+^ chelator EGTA (1 mM). As shown in Figure 4, removal of extracellular Ca^2+^ significantly blunted the effect of carmustine on annexin V binding. However, in the absence of extracellular Ca^2+ ^the percentage annexin V binding erythrocytes was still slightly, but significantly, increased by carmustine treatment (Figure 4). Thus, carmustine induced cell membrane scrambling was, mainly but not completely, dependent on the presence of extracellular Ca^2+^. 

The present study explored whether carmustine triggers eryptosis, the suicidal death of erythrocytes. The results reveal that carmustine treatment of erythrocytes drawn from healthy volunteers is followed by erythrocyte shrinkage and by breakdown of PS asymmetry of the cell membrane, both hallmarks of eryptosis. The concentrations required for the stimulation of eryptosis were well in the range of the plasma concentrations encountered following *in vivo* application of carmustine [83]. When rats were given 12 mg/kg of carmustine i.p., the peak plasma concentration approached 28 µM [83]. The elimination half-time was about 16 min [83]. At least in theory, the effect of carmustine could be shared by other nitrosourea compounds. 

The erythrocyte shrinkage following carmustine treatment is most likely the result of increased cytosolic Ca^2+^ activity, which activates Ca^2+^ sensitive K^+^ channels [20,84] leading to cell membrane hyperpolarization. The increased electrical driving force drives Cl exit and thus leads to cellular loss of KCl with osmotically obliged water [21].

The breakdown of PS asymmetry of the erythrocyte cell membrane was significantly blunted in the absence of extracellular Ca^2+^ and was again, at least in part, due to the increase of cytosolic Ca^2+^ activity ([Ca^2+^]_i_). An increase of [Ca^2+^]_i_ is well known to stimulate cell membrane scrambling with PS translocation from the inner leaflet of the cell membrane to the outer leaflet of the cell membrane [16]. Mechanisms underlying Ca^2+^ entry include Ca^2+^ permeable nonselective cation channels involving the transient receptor potential channel TRPC6 [18]. The Ca^2+^ permeable erythrocyte cation channels are activated by oxidative stress [85], a well-known effect of carmustine [2,8].

Consequences of enhanced eryptosis include anemia. *In vivo*, eryptotic erythrocytes are mainly trapped in the spleen and thus rapidly removed from circulating blood [16]. As soon as the loss of erythrocytes by triggering of eryptosis is not matched by a similar enhancement of erythropoiesis, anemia develops [16]. During carmustine treatment the myelotoxic effect of the drug [2,8] is expected to impair erythropoiesis and thus to prevent compensatory increase of erythrocyte formation. 

Consequences of enhanced eryptosis further include adhesion of PS, exposing erythrocytes to endothelial CXCL16/SR PSO [86]. The adhesion of erythrocytes to the vascular wall could at least in theory compromise microcirculation and thus interfere with blood flow [86,87,88,89,90,91]. The effect may be compounded by the stimulating effect of PS exposure on blood clotting, which may foster the development of thrombosis [87,92,93]. 

## 3. Experimental Section

### 3.1. Erythrocytes, Solutions and Chemicals

Leukocyte depleted erythrocytes were kindly provided by the blood bank of the University of Tübingen. The study is approved by the ethics committee of the University of Tübingen (184/2003V). Erythrocytes were incubated *in vitro* at a hematocrit of 0.4% in Ringer solution containing (in mM) 125 NaCl, 5 KCl, 1 MgSO_4_, 32 *N*-2-hydroxyethylpiperazine-*N*-2-ethanesulfonic acid (HEPES), 5 glucose, 1 CaCl_2_; pH 7.4 at 37 °C for 48 h. Where indicated, erythrocytes were exposed to carmustine (Enzo, Lörrach, Germany) at the indicated concentrations. Carmustine was dissolved in 50% ethanol. The final concentration of ethanol did not exceed 0.1%. Annexin V binding was not significantly different in the absence (1.8% ± 0.1%, *n* = 4) and presence of 0.1% ethanol (1.9% ± 0.2%, *n* = 4). In Ca^2+^ free Ringer solution, 1 mM CaCl_2_ was substituted by 1 mM glycol bis(2-aminoethylether)-*N*,*N*,*N*',*N*'-tetraacetic acid (EGTA). 

### 3.2. FACS Analysis of Annexin V Binding and forward Scatter

After incubation under the respective experimental condition, 50 µL cell suspension was washed in Ringer solution containing 5 mM CaCl_2_ and then stained with Annexin V FITC (1:200 dilution; ImmunoTools, Friesoythe, Germany) in this solution at 37 °C for 20 min under protection from light. In the following, the forward scatter (FSC) of the cells was determined, and annexin V fluorescence intensity was measured in FL 1 with an excitation wavelength of 488 nm and an emission wavelength of 530 nm on a FACS Calibur (BD, Heidelberg, Germany).

### 3.3. Measurement of Intracellular Ca^2+^

After incubation erythrocytes were washed in Ringer solution and then loaded with Fluo 3/AM (Biotium, Hayward, CA, USA) in Ringer solution containing 5 mM CaCl_2_ and 2 µM Fluo 3/AM. The cells were incubated at 37 °C for 30 min and washed twice in Ringer solution containing 5 mM CaCl_2_. The Fluo 3/AM loaded erythrocytes were resuspended in 200 µL Ringer. Then, Ca^2+^ dependent fluorescence intensity was measured in fluorescence channel FL 1 in FACS analysis.

### 3.4. Measurement of Hemolysis

For the determination of hemolysis the samples were centrifuged (3 min at 400*g*, room temperature) after incubation, and the supernatants were harvested. As a measure of hemolysis, the hemoglobin (Hb) concentration of the supernatant was determined photometrically at 405 nm. The absorption of the supernatant of erythrocytes lysed in distilled water was defined as 100% hemolysis.

### 3.5. Statistics

Data are expressed as arithmetic means ± SEM. As indicated in the figure legends, statistical analysis was made using ANOVA with Tukey’s test as post-test and *t* test as appropriate. *n* denotes the number of different erythrocyte specimens studied. Since different erythrocyte specimens used in distinct experiments are differently susceptible to triggers of eryptosis, the same erythrocyte specimens have been used for control and experimental conditions.

## 4. Conclusions

Exposure of erythrocytes from healthy volunteers to carmustine triggers Ca^2+^ entry with subsequent eryptosis, the suicidal erythrocyte death. Enhanced eryptosis may contribute to the development of anemia following carmustine treatment.
